# Sociality Affects REM Sleep Episode Duration Under Controlled Laboratory Conditions in the Rock Hyrax, *Procavia capensis*

**DOI:** 10.3389/fnana.2017.00105

**Published:** 2017-11-16

**Authors:** Nadine Gravett, Adhil Bhagwandin, Oleg I. Lyamin, Jerome M. Siegel, Paul R. Manger

**Affiliations:** ^1^School of Anatomical Sciences, Faculty of Health Sciences, University of the Witwatersrand, Johannesburg, South Africa; ^2^Department of Psychiatry, School of Medicine, University of California, Los Angeles, Los Angeles, CA, United States; ^3^Brain Research Institute, Neurobiology Research, Sepulveda VA Medical Centre, Los Angeles, CA, United States

**Keywords:** REM, NREM, SWA, rock hyrax, Hyracoidea, Afrotheria

## Abstract

The rock hyrax, *Procavia capensis*, is a highly social, diurnal mammal. In the current study several physiologically measurable parameters of sleep, as well as the accompanying behavior, were recorded continuously from five rock hyraxes, for 72 h under solitary (experimental animal alone in the recording chamber), and social conditions (experimental animal with 1 or 2 additional, non-implanted animals in the recording chamber). The results revealed no significant differences between solitary and social conditions for total sleep times, number of episodes, episode duration or slow wave activity (SWA) for all states examined. The only significant difference observed between social and solitary conditions was the average duration of rapid eye movement (REM) sleep episodes. REM sleep episode duration was on average 20 s and 40 s longer under social conditions daily and during the dark period, respectively. It is hypothesized that the increase in REM sleep episode duration under social conditions could possibly be attributed to improved thermoregulation strategies, however considering the limited sample size and design of the current study further investigations are needed to confirm this finding. Whether the conclusions and the observations made in this study can be generalized to all naturally socially sleeping mammals remains an open question.

## Introduction

Sleep is a homeostatically regulated process and it is characterized by its easy reversibility, immobility and reduced responsiveness to sensory stimuli. Species specific sleep postures and sleep sites, as well as closure of the eyes, are typically regarded as signs of behavioral sleep. Mammalian sleep is divided into non rapid eye movement sleep (NREM), which is often but not always synonymous to slow wave sleep (SWS), and rapid eye movement sleep (REM; Nicolau et al., 2000; Zepelin, 2005; Cirelli and Tononi, 2008; Lesku et al., 2008; Siegel, 2008). In addition to the classic mammalian sleep states an additional state, unique to the rock hyrax, termed somnus innominatus (meaning sleep without a name, SI) has been identified. It is currently not known if SI is form of low-voltage SWS or REM sleep as it is characterized by a low-voltage, high frequency electroencephalogram (EEG), an electromyogram (EMG) that is similar in amplitude to the preceding SWS episode and a mostly regular heart rate (see Gravett et al., 2012).

Many studies performed in mammals in laboratories to date have been conducted on animals housed in solitary, or asocial, conditions, even though that particular animal may be naturally social (McNamara et al., 2008). It has been suggested that animals in a social setting sleep less, exhibit more fragmented sleep patterns, and have lower NREM and REM quotas (Capellini et al., 2008a,b, 2009). A possible reason for this is that species that sleep socially can enter deeper stages of sleep, as they have the security of sleeping in a group, and are thus able to sleep more efficiently and acquire the benefits of sleep in a shorter time frame. It has also been hypothesized that social species have to invest more time in social interactions and relationships, which in effect leaves them with less time to sleep (Capellini et al., 2009).

In primates, it has been proposed that hierarchy may play a role in the manifestation of the sleep patterns that are observed (Noser et al., 2003). Male Gelada baboons show no correlation between sleep duration and social rank, whereas females and juveniles exhibit an increase in sleep duration with decreasing rank (Noser et al., 2003). Furthermore, dominant male Gelada baboons have an increased amount of transitional sleep, which indicates that increasing rank leads to a decreased amount of relaxed sleep, which may in turn lead to an increased degree of vigilance, enabling these animals to react swiftly to nocturnal dangers. This study also indicated that no correlation existed between sleep fragmentation and social rank (Noser et al., 2003). It has also been shown that group or network size in primates does not correlate with sleep times (Nunn et al., 2010); however, when *Drosophila* was placed in a socially enriched environment they exhibited an increase in daytime, but not night time, sleep (Ganguly-Fitzgerald et al., 2006). Studies by Meerlo et al. (1997, 2001) have also shown that in rodents, social conflict affects NREM sleep by increasing electroencephalographic slow wave activity (SWA) during the subsequent sleeping bout. Sleep duration was not affected by the social stimulus, but the rodents appeared to compensate for the socially induced sleep debt by increasing SWA during NREM. It was also noted that this increase in SWA was not only the result of the length of the preceding waking episode, but also the social nature of the preceding waking episode.

Despite these previous studies, to the authors’ knowledge, no comparative physiologically monitored sleep studies have been undertaken on the same animal under freely interacting social and solitary conditions. Thus, in the present study sleep was telemetrically recorded in the rock hyrax, *Procavia capensis*, under both solitary and freely interacting social conditions, extending our previous study of sleep in the rock hyrax (Gravett et al., 2012). Rock hyraxes are naturally diurnal mammals that live in colonies on rocky outcrops with crannies and crevices in which they shelter. The structure of the colonies is hierarchical consisting of a dominant male and female. The size of the colonies varies and is dependent on habitat and food availability but typically ranges between four and eight individuals. Rock hyraxes are often seen basking in the sun in the wild and have been reported to be poor thermoregulators. It has also been reported that rock hyraxes frequently heap and huddle together in captivity in an effort to conserve energy whilst in the wild many postures are adopted while resting, the most common being sitting with the head held into the body (Smithers, 1983).

The aim of the present study was thus to determine whether any significant differences existed in sleep and wake states between the social and solitary conditions to test hypotheses regarding the effect of sleeping socially.

## Materials and Methods

A total of five adult rock hyraxes, with body masses ranging between 1.74 kg and 4.3 kg (Table 1; body mass was used to identify adult individuals based on data provided in Skinner and Chimimba, 2005), were used in the present study. Permits from the Limpopo and Gauteng Provincial Governments were obtained for the capture and transport of the animals from the wild. All animals were treated and used according to the guidelines of the University of the Witwatersrand Animal Ethics Committee (clearance number: AESC 2005/8/5), and the study was approved by the University of the Witwatersrand Animal Ethics Screening Committee which parallel those of the NIH for the care and use of animals in scientific experimentation. The methods describe below are the same as those reported in Gravett et al. (2012).

**Table 1:** Species averages for total state time (A), number of episodes (B), episode duration (C) and slow wave activity (D) for each of the physiologically defined as well as the total state time for each of the behaviorally defined states (E) for the 24 h, light and dark periods for solitary and social conditions.

	24 h period				12 h light period				12 h dark period			
	Wake	SWS	SI	REM	Wake	SWS	SI	REM	Wake	SWS	SI	REM
**A. Total state time (%)**												
* Solitary*	66.8 ± 4.0	24.8 ± 2.9	3.1 ± 0.7	0.4 ± 0.2	67.3 ± 3.5	25.1 ± 2.6	3.0 ± 0.6	0.4 ± 0.2	66.1 ± 5.1	24.4 ± 3.5	3.3 ± 0.8	0.4 ± 0.3
* Social*	66.8 ± 5.0	25.7 ± 4.3	2.8 ± 0.5	0.6 ± 0.2	68.7 ± 3.3	26.7 ± 3.6	2.7 ± 0.5	0.5 ± 0.3	64.0 ± 6.4	25.3 ± 6.1	2.9 ± 0.6	0.64 ± 0.1
**B. Number of episodes**												
* Solitary*	129 ± 7	116 ± 8	27 ± 6	4 ± 2	68 ± 5	61 ± 5	13 ± 2	2 ± 1	60 ± 3	55 ± 4	14 ± 3	2 ± 1
* Social*	122 ± 9	113 ± 13	26 ± 5	5 ± 2	62 ± 5	60 ± 6	12 ± 2	3 ± 1	60 ± 7	53 ± 10	14 ± 3	2 ± 1
**C. Episode duration (s)**												
* Solitary*	448 ± 46	185 ± 17	99 ± 6	78 ± 4	429 ± 48	177 ± 12	99 ± 7	83 ± 6	467 ± 53	193 ± 23	98 ± 7	74 ± 15
* Social*	476 ± 67	196 ± 17	94 ± 5	98 ± 8	478 ± 48	191 ± 16	96 ± 4	84 ± 7	478 ± 19	201 ± 19	92 ± 7	114 ± 31
**D. Slow wave activity (mV)**												
* Solitary*	4.9 ± 0.1	9.3 ± 0.04	3.9 ± 0.1	2.4 ± 0.3	4.9 ± 0.2	9.3 ± 0.1	4.1 ± 0.2	2.6 ± 0.3	4.9 ± 0.1	9.3 ± 0.1	3.8 ± 0.2	2.5 ± 0.2
* Social*	4.6 ± 0.3	8.7 ± 0.4	3.9 ± 0.3	2.9 ± 0.5	4.4 ± 0.3	8.7 ± 0.4	3.8 ± 0.3	2.7 ± 0.5	4.7 ± 0.3	8.7 ± 0.4	4.0 ± 0.3	3.1 ± 0.5
	**Immobile**	**Quiet wake**	**Active wake**	**Eating and drinking**	**Immobile**	**Quiet wake**	**Active wake**	**Eating and drinking**	**Immobile**	**Quiet wake**	**Active wake**	**Eating and drinking**
**E. Behavior (%)**												
* Solitary*	67.9 ± 2.4	22.9 ± 3.0	5.4 ± 1.1	2.9 ± 0.3	71.7 ± 2.6	22.5 ± 3.0	3.3 ± 1.5	1.7 ± 0.7	64.2 ± 3.5	23.3 ± 3.4	7.5 ± 1.3	4.2 ± 0.5
* Social*	71.0 ± 2.3	21.0 ± 1.8	3.3 ± 0.7	3.3 ± 0.4	75.0 ± 3.7	19.0 ± 1.4	2.0 ± 1.1	2.0 ± 1.1	68.0 ± 3.3	23.0 ± 3.1	4.0 ± 0.6	4.0 ± 0.8

*All values expressed as mean ± SER*.

The animals were captured at random in groups of three from wild populations and thereafter allowed to acclimatize for a period of 1 month to the recording enclosures that had a 12:12 lighting schedule (light intensity 420 lx, measured with a digital lux meter) with temperature maintained between 19°C and 21°C. Following acclimatization one animal in the group of three that were housed together was selected randomly and implanted with a telemetric recording device (Data Sciences International) that allowed EEG, EMG and electrocardiography (ECG) recording without cables or restraint. The remaining two animals were moved into a neighboring enclosure, within the same room, that was identical to the recording/acclimatized enclosure which allowed for the recording of sleep in a solitary setting from the implanted animal continuously for 72 h (see Gravett et al., 2012). Following the solitary recording phase, the non-implanted animals were moved back into the recording enclosure with the implanted animal. The animals were then allowed an additional 3 days of acclimatization to the social setting after which sleep was recorded continuously for 72 h from the same implanted animal under social conditions. The animals were disturbed only once a day for approximately 5 min at the same time during each of the recording days for feeding.

The enclosure in which recording occurred was 1.8 × 1.5 m with a painted concrete surface that was covered with straw. The height of the chamber was approximately 1.5 m and steel mesh was placed over the top of the enclosure to prevent the animals from escaping. A wooden box (90 × 90 × 30 cm) with a Perspex roof and two entrances was placed inside the chamber and food (combinations of cucumber, tomato, sweet potato, pumpkin, apples and rabbit pellets as a source of roughage) and fresh water were supplied daily. Behavior was recorded with a low light CCD digital camera connected to a DVD recorder.

### Surgical Procedure

After acclimatization, surgical implantation of the telemetric recording device was performed. The animals were weighed before surgery and anesthetized with weight-appropriate doses of a 2:1 mixture of ketamine and xylazine (Anaket-V and Chanazine 2% Injection, Bayer HealthCare). The head and neck, left thoracic (two 2 × 1 cm) and abdominal (10 × 10 cm) regions were shaved and cleaned with chlorhexidine disinfectant (CHX, 0.5% chlorhexidine digluconate in 75% alcohol, Kyron Laboratories Pty Ltd.) before surgery commenced. These areas correspond to the regions where the EEG, EMG and ECG electrodes and telemeter would be implanted. The animal was placed on a heat blanket in order to maintain a constant body temperature throughout the surgery and the head was placed in a stereotaxic frame to prevent movement and allow for the accurate placement of the EEG and EMG electrodes. During the surgical procedure the animal was kept under a constant state of anesthesia by means of isoflurane ventilation (1%–2% in an oxygen/70% nitrous oxide mixture, isoflurane inhalation anesthetic, Safe Line Pharmaceuticals Pty Ltd.). The animal’s heart rate, body temperature and percentage oxygen saturation were monitored throughout the surgery.

Under aseptic conditions, a midsagittal incision was made over the skull and the skin and temporal muscle were reflected to expose the part of the skull overlying the motor cortex. Using a dental drill, three 2-mm-diameter holes were made in the cranial vault to expose the underlying dura mater. The first hole was drilled anterior to the olfactory bulbs for the placement of the indifferent electrode, while two holes were drilled approximately 5 mm apart just lateral to the sagittal sinus over the left motor cortex for the placement of the stainless-steel recording electrodes (gauge of electrode 0.457 mm, silastic outside diameter 0.9 mm and inside diameter 0.508 mm, PhysioTel^®^ Multiplus Transmitter, Data Sciences International). The electrodes were placed in such a manner that the tips rested firmly on the surface of the cortex and were secured in place with dental cement. Two of the stainless-steel EMG electrodes (1.5 cm apart) were sutured into the dorsal nuchal musculature, while two ECG electrodes (3 cm apart) were sutured into the subdermal tissue overlying the left thoracic region. A subcutaneous pocket was created (10 × 10 cm) over the left abdominal region for the implantation of the telemetry unit. All skin incisions were sutured following implantation. After surgery was complete, the animal was given an intramuscular analgesic (0.1 ml Tamgesic, Schering-Plough, mixed with 0.9 ml sterile water, 1 ml mixture/kg) and returned to the recording enclosure. Recovery was monitored every half hour until it could be established that the animal was able to move freely and eat and drink normally.

### Sleep Recording

After the surgical procedure the animal was allowed a recovery period of 1 week before the recording of sleep commenced. The animals were housed in the same enclosure (i.e., the enclosure they were acclimatized to prior to surgery and experimentation) within a sound-attenuating room for recovery as well as recording. A receiver was mounted and secured to one wall of the enclosure while a low light CCD digital camera was mounted above the enclosure. The telemetric recording system (Data Sciences International, DSI, PhysioTel Multiplus Transmitter, model TL10M3-D70-EEE implant—this particular model provided the strongest signal in our recording enclosure, smaller transmitters were unable to transmit a signal to the receiver as the distance over which it had to operate was too long) consisted of a DEM multiplex interface to which the receiver was connected. The signal from the implanted transmitter (round, 13 cm^2^ with stainless steel electrodes, weight 37 g, volume 25 ml, 3 channels) detected by the receiver was relayed to the input amplifier of the Data Sciences computer system, after which it was digitally recorded (in DSI format) for analysis. After the recording was completed, data digitally saved in the DSI format was converted to text format and these files were in turn converted into the appropriate format needed for recognition and analysis by the Spike 2 computer program (version 4.2, Cambridge Electronic Design).

### Data Analysis

**Figure 1:**
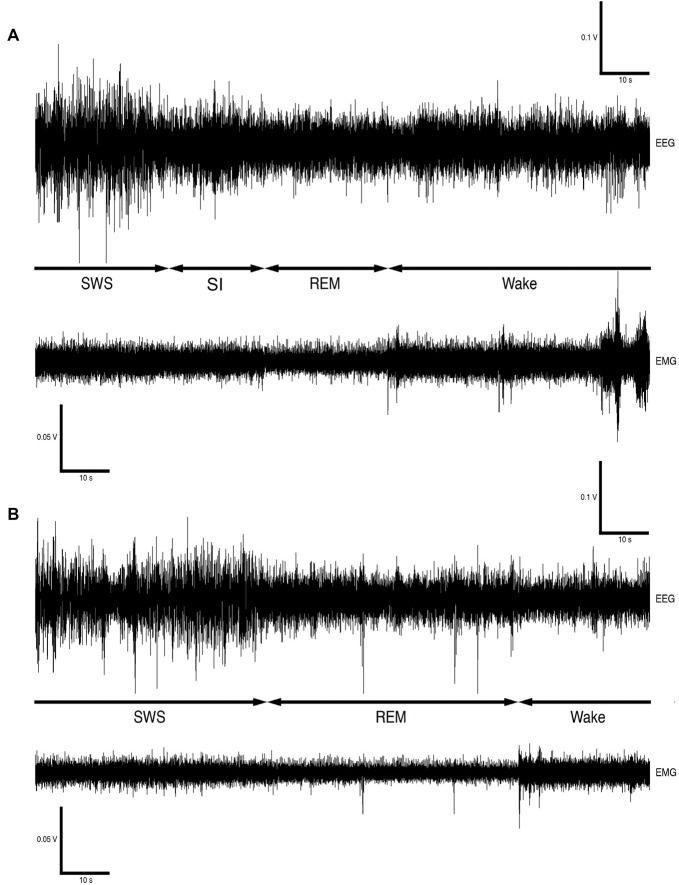
Electroencephalogram (EEG) and electromyogram (EMG) polygraphs (**A,B**, from Gravett et al., 2012; Copyright © 2012 Krager Publishers, Basel, Switzerland) illustrating state transitions. The first set of polygraphs **(A)** illustrates the transition from SWS → SI → REM → wake while the second set of polygraphs **(B)** illustrates the transition from SWS → REM → wake.

Version 4.2.2 of the Spike 2 software (Cambridge Electronic Designs, UK) was used in order to convert the recorded data into the appropriate format, i.e., Spike 2 data format, for offline analysis. The EEG data was scored in 5-s epochs as: (1) wake, characterized by low-voltage, high-frequency EEG and high-voltage EMG; (2) SWS, characterized by high-voltage, low-frequency EEG and EMG lower in amplitude than waking; (3) REM, characterized by low-voltage, high-frequency EEG, an almost atonic EMG and irregular ECG, or (4) somnus innominatus (SI), characterized by a low-voltage, high-frequency EEG, EMG amplitude characteristic of SWS and a regular ECG (Gravett et al., 2012; Figure 1). An epoch was only assigned to a particular state if the state occupied at least 50% of the epoch. The modal state per minute was calculated from the 5-s epoch data and was used in all further analyses. The power spectrum for each of the defined states was calculated with the Spike 2 computer program (Hanning window, FFT number 512, sampling frequency 500 Hz, segment length 1.024 s; see Gravett et al., 2012) and converted from mV^2^ to mV which was used in all subsequent statistical analyses.

Behavior was scored in 1-min epochs as: (1) immobile—animal was completely immobile for >30 s; (2) quiet waking—animal was immobile and only moving its head or made minor movements in the same place for >30 s; (3) active waking—animal was actively moving around for >30 s (this state included exploratory and grooming behavior); or (4) eating/drinking—animal was eating and/or drinking for >30 s.

Data was tested for normality prior to statistical analysis (Shapiro-Wilk’s W test for normality, *p* > 0.05). All data was normally distributed and there was no need for data transformation. A *t*-test for dependent variables was used in all statistical analyses and a significant difference was obtained in all cases with *p* < 0.05. Statistical tests were performed to determine whether significant differences existed between the solitary and social settings with regard to total state times, number of episodes, and episode duration for the 24 h, light and dark periods. The dependent *t*-test was also used to determine whether significant differences existed between the solitary and social setting with regard to sleep cycle length, SWA during SWS and during all states as well as the behaviorally defined states for the 24 h, light and dark periods. The Pearson correlation test was used to determine the degree of correlation between the behavior of the implanted and non-implanted animals (significant correlation, *p* < 0.05). Microsoft Excel, Minitab 17 and GraphPad Prism 6 were used in the scoring, analysis and graphing of the data.

## Results

The physiologically measurable parameters of sleep as well as the associated behaviors were recorded in a total of five hyraxes continuously for a period of 72 h under solitary conditions (Gravett et al., 2012). This was followed by the introduction of one or two other non-implanted hyraxes to the enclosure of the implanted hyrax, followed by 72 h of continuous recording under social conditions. The polygraphic data was scored in 5 s epochs as wake, SWS, SI or REM (Figure 1) and the modal state per minute was determined and used in all further analyses (Gravett et al., 2012). There was no significant difference between solitary and social conditions for total state times, number of episodes, episode duration or SWA for all states. A significant difference was however observed for REM sleep duration. REM sleep duration was on average 20 s longer daily and 40 s longer during the dark period under social conditions (Table 1). For brevity, only the social condition results are reported in the sections that follow. Please refer to Tables 1, 2 for a complete summary of all mean ± SER values for both solitary and social conditions for all periods.

**Table 2:** Average sleep cycle length (A) and individual state contributions to the sleep cycle (B) including and excluding wake episodes, and state occurrences before and after REM sleep episodes (C).

**A. Sleep cycle length (min)**							
	**Including wake episodes**				**Excluding wake episodes**		
* Solitary*	238.6 ± 55.7				64.5 ± 13.1		
* Social*	222.8 ± 38.9				66.7 ± 10.8		
**B. State contribution to sleep cycle (%)**							
	**Including wake episodes**				**Excluding wake episodes**		
	**Wake**	**SWS**	**SI**	**REM**	**SWS**	**SI**	**REM**
* Solitary*	74.4	22.3	2.8	0.4	87.6	11.2	1.4
* Social*	68.5	28.0	2.9	0.9	87.5	9.8	3.2
**C. State occurance (%)**							
	**Before REM sleep episodes**				**After REM sleep episodes**		
	**Wake**	**SWS**	**SI**		**Wake**	**SWS**	**SI**
* Solitary*	1.5	51.2	47		62.1	30.3	7.6
* Social*	7.9	41.3	50.8		69.8	28.6	1.6

*All values expressed as mean ± SER*.

### Time Budgets

**Figure 2:**
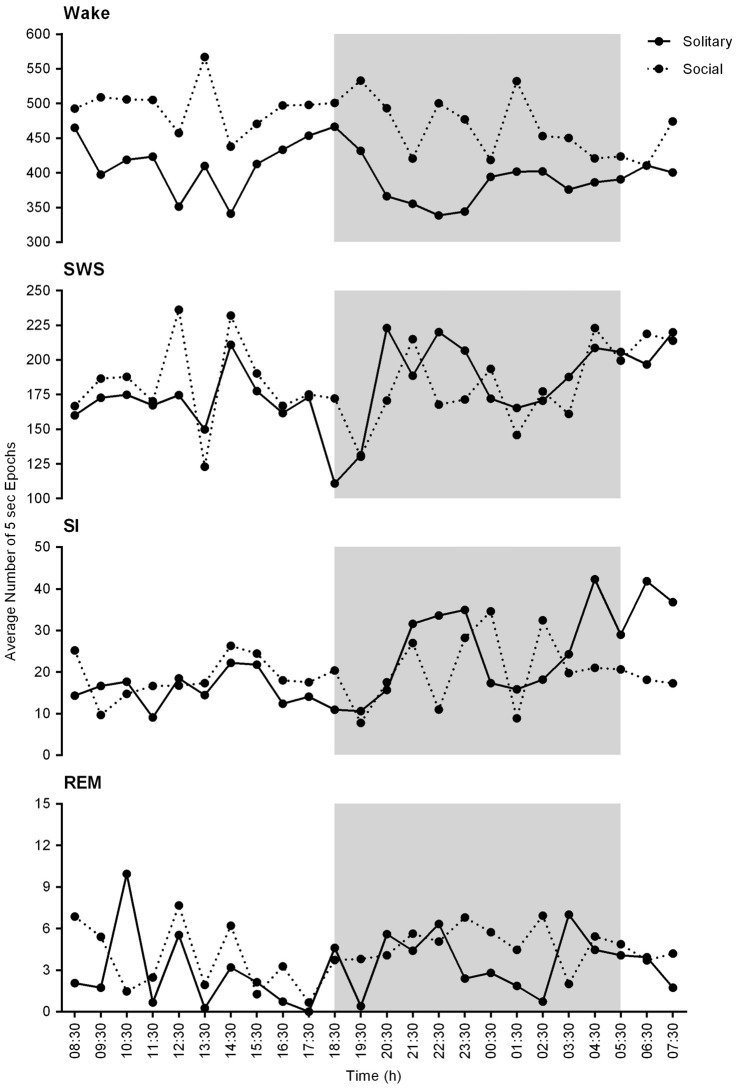
Physiological state correlations between solitary (**solid lines**) and social (**dotted lines**) conditions. These graphs represent the average number of 5 s epochs per hour for each of the defined physiological states over the 24 h period. No significant differences were noted between solitary and social conditions. In general, the average number of epochs for each of the defined states is slightly increased under solitary conditions and a similar trend in the hourly distribution of each of the defined states is observed for both conditions for the 24 h period. The shaded area represents the dark period.

**Figure 3:**
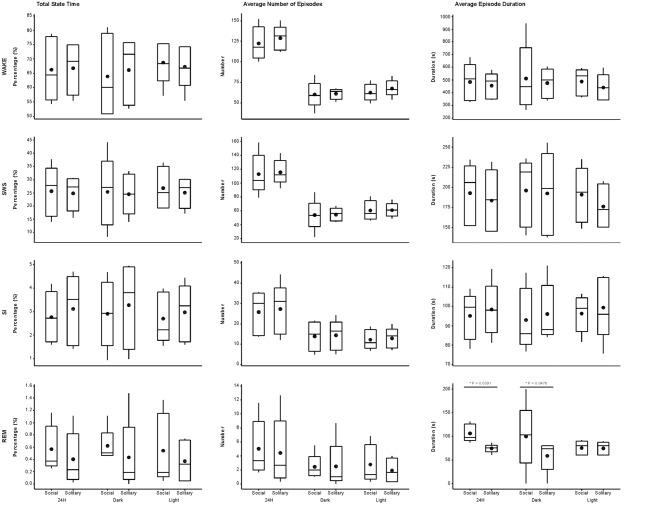
Box and whisker plots illustrating the total amount of time spent in each of the defined physiological states (**left set of graphs**), the average number of episodes (**middle set of graphs**) and episode duration (**right set of graphs**) for solitary and social conditions for the 24 h **(left set of graphs on each individual graph)**, dark **(middle set of graphs on each individual graph)** and light **(right set of graphs on each individual graph)** periods. No significant difference was noted for total state time, number of episodes and episode duration for each of the defined states for the 24 h, light and dark periods, except for REM episode duration, which was greater during social conditions for the 24 h and dark periods. Significant differences between solitary and social conditions are indicated by a star and the respective *p*-value is indicated on the graph (dependent *t*-test, *p* > 0.05, d.f. = 4, please refer to results section for respective *t*—values). For each condition, there was also no difference between the light and dark periods for total state time, number of episodes and episode duration for each of the defined states (dependent *t*-test, *p* > 0.05, d.f. = 4). The mean is shown by the circles and the median by the horizontal bar within each box.

On a daily basis, under social conditions, the percentage of time spent awake was on average 66.8% (± 16.0 h), SWS 25.7% (± 6.2 h), SI 2.8% (± 40.3 min), and REM sleep 0.6% (± 8.6 min). During the light- and dark periods, 68.7% (± 8.2 h) and 64.0% (± 7.7 h) of time was spent awake, 26.7% (± 3.2 h) and 25.3% (± 3.0 h) in SWS, 2.7% (± 19.4 min) and 2.9% (± 20.9 min) in SI, and 0.5% (± 3.6 min) and 0.64% (± 4.6 min) in REM sleep, respectively. These values did not differ significantly from the daily, light- or dark period results obtained under solitary conditions (dependent *t*-test, *p* > 0.05, d.f. = 4 in all instances; Table 1, Figures 2, 3).

### Number of Episodes

The daily average number of wake-, SWS-, SI- and REM sleep episodes under social conditions amounted to 122, 113, 26 and 5, respectively. During the light period, the average number of episodes for wake was 62, 60 for SWS, 12 for SI, and 3 for REM sleep. On average, the dark period consisted of 60 wake-, 53 SWS-, 14 SI-, and 2 REM sleep episodes. Like total state times, the average number of episodes for each of the defined states, for all periods, under social conditions did not differ significantly from those observed under solitary conditions (dependent *t*-test, *p* > 0.05, d.f. = 4 in all instances; Table 1, Figure 3).

### Duration of Episodes

The average duration of wake episodes under social conditions remained relatively constant across the periods (daily—476 s, light period—478 s, dark period—478 s). SWS episode duration was similar daily (196 s) and during the light (191 s) period and increased marginally during the dark (201 s) period. SI episode duration was relatively constant (daily—94 s, light period—96 s, dark period—92 s), while REM sleep episode duration showed some variation across all three periods (daily—98 s, light period—84 s, dark period—114 s). For the majority of the defined states, across all periods, social sleep episode duration did not differ significantly from those under solitary conditions (dependent *t*-test, *p* > 0.05, d.f. = 4 in all instances), however a significant difference did exist between conditions for REM sleep episode durations, daily and during the dark period (dependent *t*-test, *p* = 0.009, *t* = 3.421 (daily); *p* = 0.047, *t* = −2.824 (dark period); Table 1, Figure 3). REM sleep duration was on average approximately 20 s longer daily and 40 s longer during the dark period under social conditions.

### State Transition Probabilities

**Figure 4:**
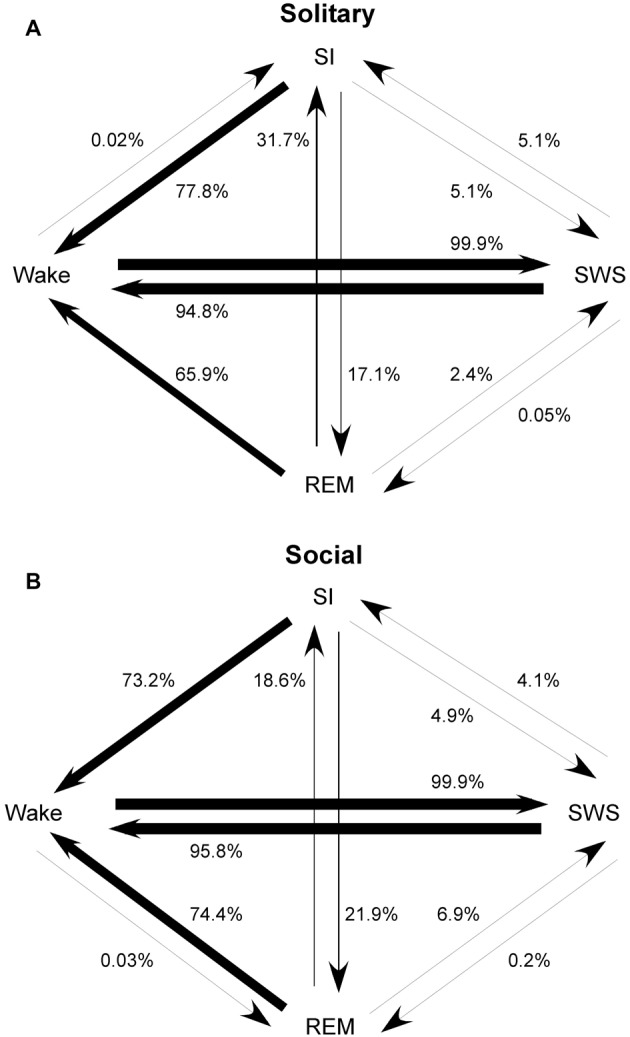
State transition probabilities in the rock hyrax for solitary (from Gravett et al., 2012; Copyright © 2012 Krager Publishers, Basel, Switzerland) **(A)** and social conditions **(B)**. The most common state transition pathway for both conditions is wake → SWS → wake. Under social conditions: (1) REM transitions more frequently to SWS and wake and less frequently to SI; (2) SI transitions more frequently to REM; (3) wake transitions to REM but not to SI. All other state transition probabilities are similar between the two conditions. The second most common state transition pathway during both conditions is wake → SWS → SI → wake.

The most common state transition pathway during both conditions was wake → SWS → wake. Under social conditions: (1) REM transitioned more frequently to SWS and wake and less frequently to SI; (2) SI transitioned more frequently to REM; (3) wake transitioned to REM but not to SI. All other state transition probabilities were similar between the two conditions. The second most common state transition pathway during both conditions was wake → SWS → SI → wake (Figure 4).

### Sleep Cycle Length and Architecture

**Figure 5:**
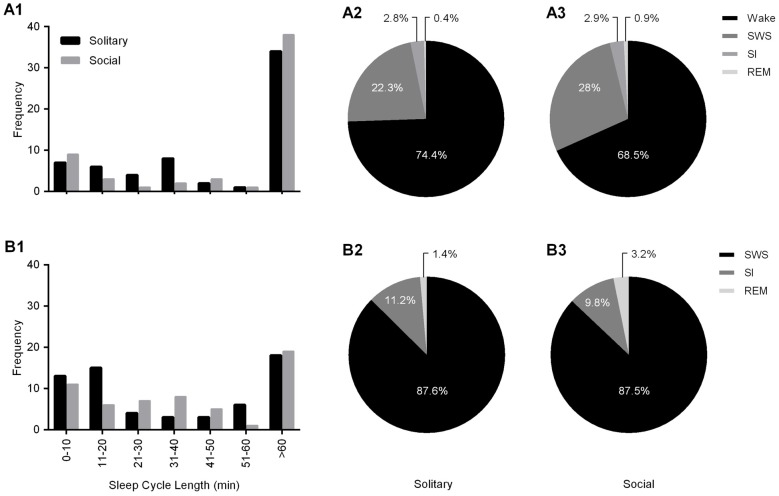
Frequency distributions of sleep cycle length including **(A1)** and excluding wake **(B1)** episodes for solitary (**black bars**) and social (**gray bars**) conditions. For both conditions, irrespective of whether wake episodes are included or excluded from the calculations, the sleep cycle length mostly exceeds 60 min. The second most common sleep cycle length ranges between 31–40 min (wake episodes included) and 11–20 min (wake episodes excluded) under solitary conditions and showed no change under social conditions, ranging between 0–10 min (including and excluding wake episodes). The Pie charts illustrate the percentage contribution of each of the physiologically defined states to the sleep cycle including **(A2,3)** and excluding **(B2,3)** wake episodes for both solitary (**left**) and social (**right**) conditions. No significant differences were noted with regard to the contribution of each of the defined states to the sleep cycle (including and excluding wake episodes) between solitary and social conditions, however it is worth noting that the contribution of REM sleep to the sleep cycle, including and excluding wake episodes is increased in the social condition by 125% and 128%, respectively.

Sleep cycle length was calculated for both conditions as the time between the onset of one REM sleep episode to the next. Due to the nature of the sleep architecture of the rock hyrax, as well as the limited amount of REM sleep observed in this species, the sleep cycle length was calculated with wake episodes included and excluded. The average sleep cycle length under social conditions with wake episodes included was 222.8 min and 66.7 min when wake episodes were excluded. No significant difference was noted between social and solitary conditions with regard to sleep cycle length, including or excluding wake episodes (dependent *t*-test, *p* > 0.05 and d.f. = 56 in all instances; Table 2, Figures 5A1,B1). The time of day during which the longest sleep cycle occurred was highly variable between days, individuals and experimental conditions.

The sleep cycle was further examined to determine the contribution of each of the defined states to the sleep cycle. When wake episodes were included as part of the sleep cycle, in the social condition, it accounted for 68.5% of the cycle composition, SWS 28%, SI 2.9% and REM sleep 0.9%. However, when wake episodes were excluded from the sleep cycle, it consisted of 87.5% SWS, 9.8% SI, and 3.2% REM sleep. No significant differences were noted between conditions with regard to the contributions of each of the defined states to the composition of the sleep cycle, but it is worth noting that the percentage contribution of REM sleep to the sleep cycle, including and excluding wake episodes increased in the social setting by 125% and 128%, respectively (Table 2, Figures 5A2,3,B2,3).

With regard to the states that most commonly preceded REM sleep in the sleep cycle, in the social condition SI preceded REM 50.8% of the time, followed by SWS (41.3%) and wake (7.9%), whereas in the solitary condition SWS preceded REM most often (51.2%) followed by SI (47%) and wake (1.5%). The states that most commonly followed REM in the sleep cycle were similar in the social and solitary conditions, with wake being most common, followed by SWS and SI (Table 2).

### Slow Wave Activity

**Figure 6:**
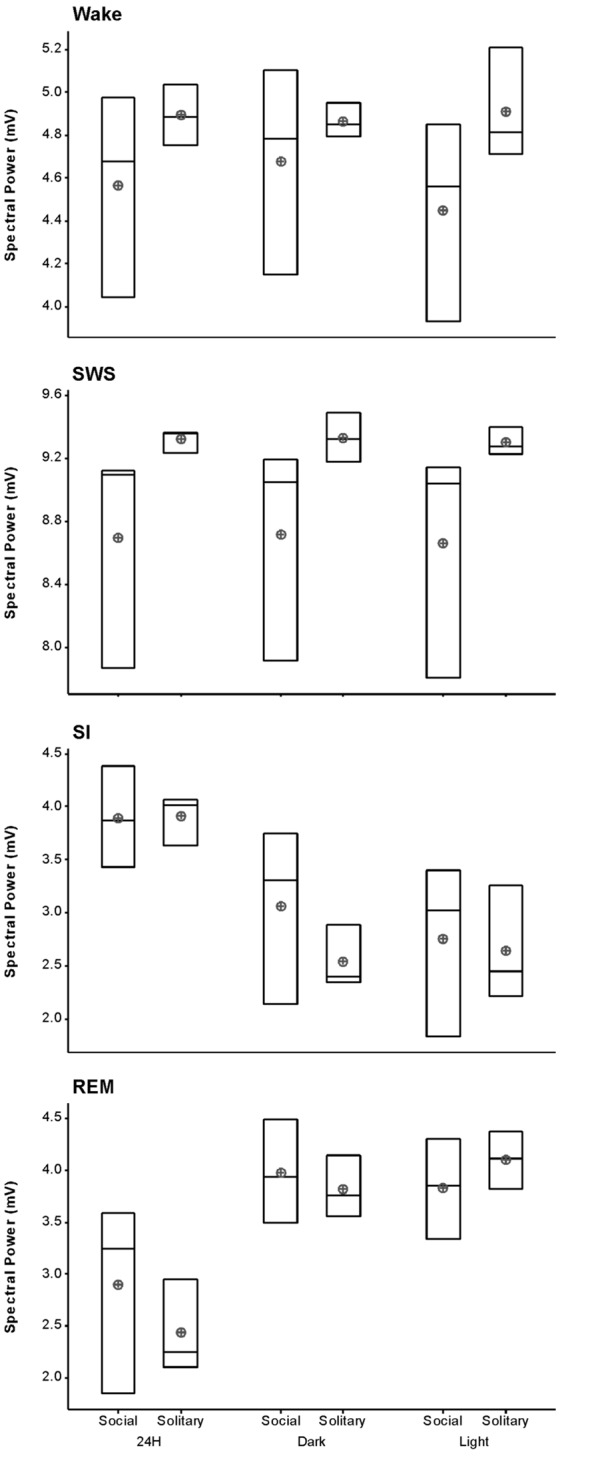
Box plots showing slow wave activity (SWA; based on 2-h intervals) for all states for the 72 h recording period for solitary and social conditions. Data presented are the daily averages of three animals. No significant differences were observed between solitary and social conditions across all periods (dependent *t*-test, *p* > 0.05, d.f. = 2). Significant differences were noted between the light and dark periods for SWA during wake, SI and REM sleep under social conditions. For each of these sates SWA was greater during the dark period (dependent *t*-test, *p* < 0.005, d.f. = 2, please refer to results section for specific *p*- and *t*-values). The mean is shown by the circles and the median by the horizontal bar within each box.

The average SWA based on 2-h intervals was calculated for SWS and all other states. Under social conditions SWA was greatest during SWS (8.7 mV) and gradually declined in intensity from wake (4.6 mV) to SI (3.9 mV) to REM sleep (2.9 mV). These results were similar to those observed under solitary conditions and no significant difference was observed between conditions for all periods (dependent *t*-test, *p* > 0.05 and d.f. = 2 in all instances; Table 1, Figure 6).

### Instantaneous Heart Rate

Instantaneous heart rate (IHR) under social conditions was on average 174 bpm (SER ± 25.2) for wake, 155 bpm (SER ± 6.9) for SWS, 156 bpm (SER ± 9.2) for SI, and 110 bpm (SER ± 14.2) for REM sleep. Under solitary conditions IHR was on average 154 bpm (SER ± 7.8) for wake, 158 bpm (SER ± 11.0) for SWS, 153 bpm (SER ± 10.6) for SI, and 163 bpm (SER ± 27.3) for REM sleep. IHR during wake and REM sleep was significantly different between conditions (dependent *t*-test, *p* = 0.000 (both), *t* = 14.85 (wake) and −18.74 (REM sleep), d.f.= 4 for both). IHR during wake was greater and more variable under social conditions, whereas IHR during REM sleep was greater and more variable under solitary conditions.

### Light vs. Dark

**Figure 7:**
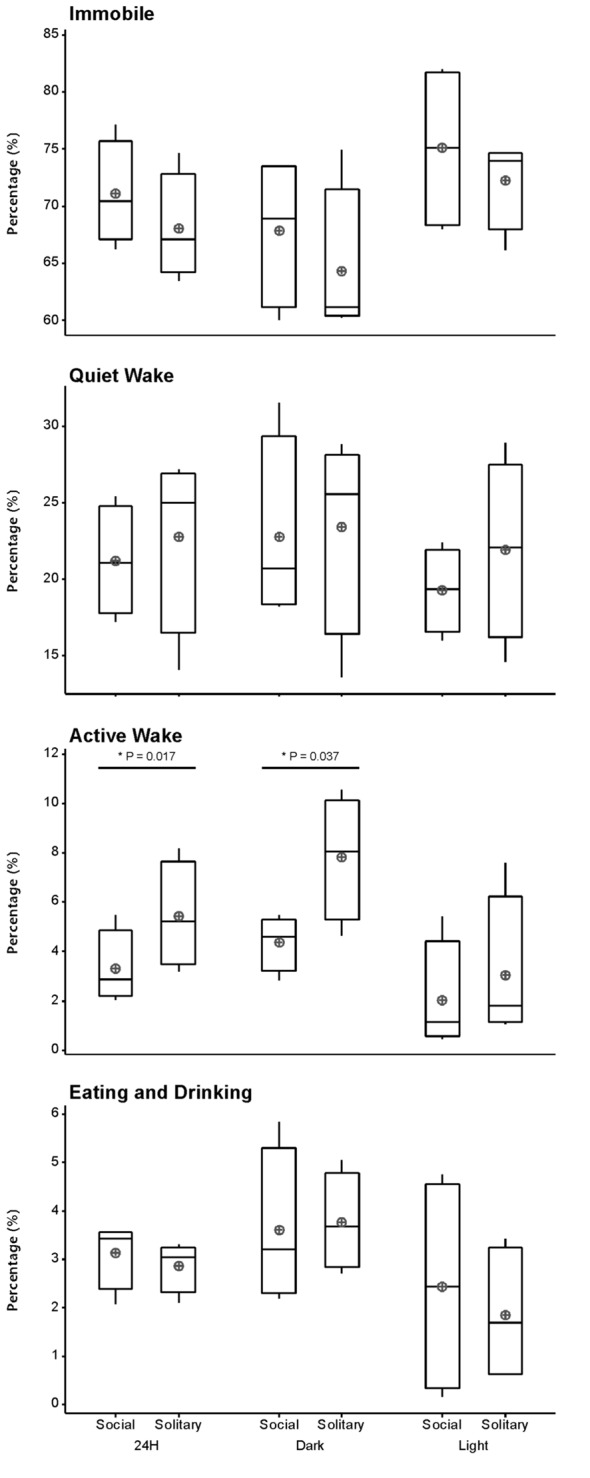
Box and whisker plots illustrating the percentage of time occupied by each behavioral state for the 24 h, light and dark periods under solitary and social conditions. All behavioral states, with the exception of active wake, showed no significant difference between solitary and social conditions. More active wake is observed under solitary conditions for the 24 h and dark periods. A star indicates significant differences between solitary and social conditions and the respective *p*-value is indicated on the graph (dependent *t*-test, *p* > 0.05, d.f. = 3, please refer to results section for respective *t*—values). Significant differences were also noted between the light and dark periods for immobility under solitary conditions and active wake under both conditions. In all instances, these behaviors were greater during the dark period (dependent *t*-test, *p* < 0.005, d.f. = 2, please refer to results section for specific *p*- and *t*-values). The mean is shown by the circles and the median by the horizontal bar within each box.

Despite the rock hyrax being naturally a diurnal mammal, under controlled laboratory conditions this does not seem to hold true. No significant difference existed between the light and dark periods for both conditions for total state times, number of episodes or episode durations (dependent *t*-test, *p* > 0.05 and d.f. = 4 in all instances; Table 1, Figure 3). A significant difference between the light and dark periods was however noted under social conditions for SWA during wake (*p* = 0.002, *t* = −20.21), SI (*p* = 0.003, *t* = −18.07) and REM sleep (*p* = 0.043, *t* = −4.67; dependent *t*-test, d.f. = 2 in all instances). SWA during the aforementioned states was greatest during the dark period compared to the light period (Table 1, Figure 6). Further significant light-dark differences were noted under solitary conditions for immobile behavior (*p* = 0.03, *t* = 3.28), and active wake behavior under both conditions (social: *p* = 0.029, *t* = −3.34; solitary: *p* = 0.017, *t* = −3.96; dependent *t*-test, d.f. = 4 in all instances). Animals were more immobile during the light period under solitary conditions, whilst more active wake occurred during the dark period under both conditions (Table 1, Figure 7).

### Behavioral Data

Behavior was recorded in concert with the physiological recordings and scored in 1 min epochs as immobile, quiet wake, active wake (which included exploratory and grooming behavior) or eating and drinking. Daily, under social conditions, approximately 71% (± 17.0 h) was spent immobile, 21% (± 5.0 h) in quiet wake, 3.3% (43.2 min) in active wake, and 3.3% (43.2 min) eating and drinking. No significant differences were noted for each of the behaviorally defined states between conditions daily (dependent *t*-test, *p* > 0.05 and d.f. = 3 in all instances), with the exception of active wake which was on average 30 min longer under solitary conditions (dependent *t-test*, *p* = 0.0017, *t* = 3.464 and d.f. = 3; Table 1, Figure 7). During the light and dark periods respectively, under social conditions, 75% (± 9.0 h) and 68% (8.2 h) of the time was spent immobile, 19% (2.3 h) and 23% (2.8 h) in quite wake, 2% (14.4 min) and 4% (28.8 min) in active wake, and 2% (14.4 min) and 4% (28.8 min) eating and drinking (Table 1, Figure 7). No significant differences were noted with regard to the amount of time spent in each of the behaviorally defined states between conditions for the light and dark periods (dependent *t*-test, *p* > 0.05 and d.f. = 3 in all instances), with the exception of active wake which was on average 25 min longer during the dark period under solitary conditions (dependent *t*-test, *p* = 0.037, *t* = 3.174, and d.f. = 3; Table 1, Figure 7).

**Figure 8:**
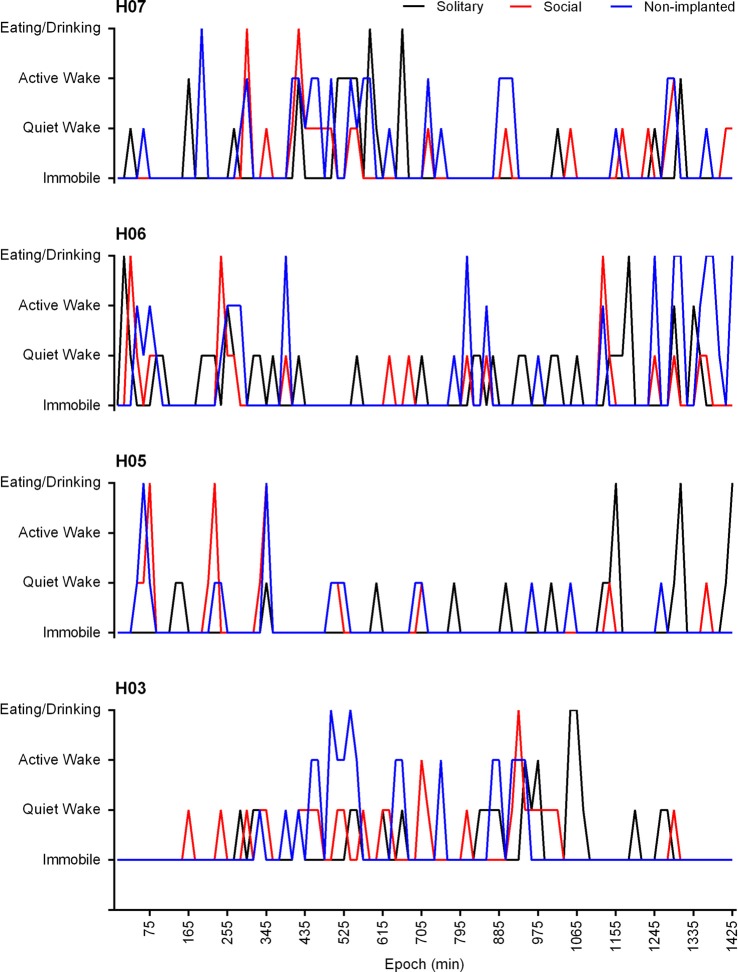
Hypnograms showing the behavioral state transitions over a 24 h period for H07, H06, H05 and H03 under solitary (**black lines**) and social (**red lines**) conditions as well as that of the non-implanted (**blue lines**) animal under social conditions. Due to the variability in the recording start times for each individual animal the time scale has been adapted to represent the number of 1 min epochs from the recording start time. The data presented is from the 3rd recording day and represents the 15 min modal behavior. There is no significant difference between the behavioral state transitions under solitary and social conditions over a 24 h period. A significant correlation exists between the behavior of the implanted and non-implanted animal/s under social conditions (Pearson’s *r* = 0.3809, *p* < 0.0001) and the behavior of the non-implanted animal shows a 72.9% agreement with that of the implanted animal.

In the social setting, the behavior of the non-implanted animals was also scored and analyzed. The non-implanted animals spent similar amounts of time immobile (79.7%), and eating and drinking (3.4%) to that reported for the implanted animals in the social and solitary conditions, but they spent less time in the quiet wake (7.6%) state and showed increased amounts of active wake (3.4%). A significant correlation (Pearson’s *r* = 0.3809, *p* < 0.0001) existed between the behavior of the implanted and non-implanted animal(s), and in all cases the probability of the behavior of the implanted vs. non-implanted animals being unrelated was found to approach zero. In addition, a 72.9% agreement was also observed with regard to the implanted and non-implanted animals entering the same behavioral state at the same time (Figure 8).

## Discussion

The aim of the present study was to investigate the effect of sociality on sleep architecture in a naturally occurring social, diurnal species, the rock hyrax. Most studies that have reported on the possible effects of sociality on the evolution of sleep in mammals have compared solitary species to social species. It has been hypothesized that social species sleep less, have more fragmented sleep and lower NREM and REM sleep quotas (Capellini et al., 2008a,b,^2009^). It is also believed that social species invest more time in social interactions, which in effect leave them with less time to sleep. According to these studies, social species can enter deeper stages of sleep and in doing so sleep more effectively in a shorter period of time (Capellini et al., 2009). As these hypotheses are primarily based on observations comparing solitary and social species and not solitary and social conditions within the same species, the results from the present study are discussed in relation to these hypotheses and cannot support or refute them.

### Total Sleep Times and Sleep Fragmentation

Our results revealed no significant difference in total sleep time or the average number of sleep episodes between conditions across all periods. In general, a negligible increase in total sleep time (combined SWS, SI and REM sleep time) was observed under social conditions while the number of sleep episodes (combined SWS, SI and REM sleep episodes) increased marginally under solitary conditions. We can thus conclude that under social conditions in a controlled laboratory environment, total sleep time does not become reduced and sleep does not become more fragmented.

### NREM and REM Sleep Quotas and Sleep Cycle Composition

NREM and REM sleep quotas are said to be reduced in social species (Capellini et al., 2009). In the current study, we did not see a significant difference between conditions across all periods for SWS and REM sleep quotas as they were only marginally increased under social conditions. The average duration of REM sleep episodes was however significantly different daily and during the dark period when the conditions were compared. The average duration of REM sleep episodes was approximately 20 s and 40 s longer under social conditions, daily and during the dark period respectively. Looking at the composition of the sleep cycle our results also revealed that the percentage contribution of REM sleep to the sleep cycle increased under social conditions by 125% when wake episodes were included, and 128% when wake episodes were excluded. It was also noted that the range of REM sleep episode duration within the sleep cycle was also greater under social conditions compared to solitary conditions.

It is possible that the increase in REM sleep duration and contribution to the sleep cycle under social conditions could be linked to improved thermoregulation. Thermoregulation in the rock hyrax involves both physiological and behavioral (i.e., basking and huddling) mechanisms. Laboratory based studies have shown that hyraxes are capable of maintaining a constant body temperature when exposed to a range of ambient temperatures that they would naturally encounter. In addition, these studies also reported that hyraxes become hypothermic at night and rely on morning solar basking as opposed to metabolic warming to increase body temperature (Taylor and Sale, 1969). Apart from solar basking, hyraxes also employ another behavioral strategy to conserve body heat, especially during the night—huddling. Energy expenditure is reduced during the sleep phase when animals huddle together as the surface area to which heat is lost to the environment is reduced (Weatherhead et al., 1985). Brown and Downs (2006) thus hypothesized that social behavior in the rock hyrax may play a significant role in thermoregulation.

It has been reported that a relationship exists between total REM sleep time and the temperature of an animal’s environment. At thermoneutral temperatures total REM sleep time is maximal, whereas heat or cold stress results in a decline. Studies have also shown that a brief change in skin temperature toward thermoneutrality is capable of triggering REM sleep in cold stressed neonatal rats (Szymusiak et al., 1980; Szymusiak and Satinoff, 1981). Thus, it is possible that the increased contribution of REM sleep to the sleep cycle as well as the increase in the average REM sleep duration, especially during the dark period, could be accounted for by improved thermoregulation during social conditions. Huddling together at night could aid in maintaining a constant body temperature throughout this period and the skin-to-skin contact could possibly activate peripheral thermoreceptors, potentially resulting in more frequent REM episodes. REM sleep is not the only state affected by raised skin temperature. A number of studies have reported that increases in skin temperature also affect NREM sleep in both humans and animals. Deeper stages of NREM sleep has been reported with as little as a 0.4°C increase in skin temperature in young and old healthy and insomniac participants, while afternoon body heating in humans and whole-body heating during the last 4 h of the light period in rats have been associated with increased NREM quotas (Morairty et al., 1993; Raymann et al., 2008; Romeijn et al., 2012). Despite these reports, our results did not show a significant increase in total SWS time or duration, as the increases seen under social conditions were negligible. As body temperature was not measured in the current study, our interpretation of the increased REM sleep duration and contribution to the sleep cycle as a possible result of improved thermoregulation under social conditions, is highly speculative and requires further investigation.

### SWA and Sleep Efficiency

Another hypothesis related to sleep in social species proposes that socially sleeping species enter deeper stages of sleep and thus sleep more efficiently over shorter periods of time. This need to maximize sleep efficiency in a shorter time frame is believed to be due to a trade-off that exists between times devoted to social interactions and sleep—more time is invested in social interactions leaving less time for sleep (Capellini et al., 2009). SWA during NREM is considered a measure of sleep intensity and sleep debt, as well as an indicator of NREM sleep homeostasis (Borbély and Neuhaus, 1980; Daan et al., 1984; Tobler and Borbély, 1986; Meerlo et al., 1997, 2001; Borbély and Achermann, 1999). SWA is usually highest during the first NREM sleep episode and decreases during later NREM sleep episodes. The intensity of NREM SWA is reportedly also influenced by the duration as well as the nature of the prior wake experience (Bellesi et al., 2014). Sleep deprivation, intensive learning, social defeat and stress during the preceding wake experience can all lead to an increase in NREM SWA (Meerlo et al., 1997, 2001; Bellesi et al., 2014; Kamphuis et al., 2015). As our results showed no significant difference in SWA for all states between conditions across all periods we can conclude that sleep efficiency does not appear to be significantly enhanced under social conditions in the rock hyrax.

## Conclusion

The current study provides four results of interest regarding the effects of sleeping in a social setting for the rock hyrax: (1) total sleep times remain similar to solitary conditions; (2) sleep does not become more fragmented; (3) sleep efficiency remains unchanged; and (4) REM sleep episode duration and overall contribution to the sleep cycle increases. The significant increase in REM episode durations and increased contribution to the sleep cycle could be a function of improved thermoregulation under social conditions, however this is highly speculative and requires further investigation. Based on these findings, it is possible to assume that sleep quality does not improved significantly under social conditions in the rock hyrax, however considering the limited sample size and design of the current study further investigations are needed to confirm this finding. Whether the conclusions and the observations made in this study can be generalized to all naturally socially sleeping mammals remains an open question.

## Author Contributions

NG and PRM conceptualized the study. PRM obtained the funding. NG, PRM, AB and OIL performed the experimental procedures. NG analyzed the data and wrote the manuscript. PRM, AB, OIL and JMS contributed to the editing and improvement of drafts of the manuscript. All authors had full access to all of the data in the study and take responsibility for the integrity of the data and the accuracy of the data analysis.

## Conflict of Interest Statement

The authors declare that the research was conducted in the absence of any commercial or financial relationships that could be construed as a potential conflict of interest.
